# Effects of Different Cooling Treatments on Heated Granite: Insights from the Physical and Mechanical Characteristics

**DOI:** 10.3390/ma17184539

**Published:** 2024-09-15

**Authors:** Qinming Liang, Gun Huang, Jinyong Huang, Jie Zheng, Yueshun Wang, Qiang Cheng

**Affiliations:** 1 School of Resources and Safety Engineering, Chongqing University, Chongqing 400044, China; 2 School of Geology and Mining Engineering, Xinjiang University, Urumqi 830046, China

**Keywords:** rock mechanical properties, cooling treatments, heated granite, experimental research

## Abstract

The exploration of Hot Dry Rock (HDR) geothermal energy is essential to fulfill the energy demands of the increasing population. Investigating the physical and mechanical properties of heated rock under different cooling methods has significant implications for the exploitation of HDR. In this study, ultrasonic testing, uniaxial strength compression experiments, Brazilian splitting tests, nuclear magnetic resonance (NMR), and scanning electron microscope (SEM) were conducted on heated granite after different cooling methods, including cooling in air, cooling in water, cooling in liquid nitrogen, and cycle cooling in liquid nitrogen. The results demonstrated that the density, *P*-wave velocity (*V*_p_), uniaxial compressive strength (UCS), tensile strength (*σ*_t_), and elastic modulus (*E*) of heated granite tend to decrease as the cooling rate increases. Notably, heated granite subjected to cyclic liquid nitrogen cooling exhibits a more pronounced decline in physical and mechanical properties and a higher degree of damage. Furthermore, the cooling treatments also lead to an increase in rock pore size and porosity. At a faster cooling rate, the fracture surfaces of the granite transition from smooth to rough, suggesting enhanced fracture propagation and complexity. These findings provide critical theoretical insights into optimizing stimulation performance strategies for HDR exploitation.

## 1. Introduction

With the increasing global demand for energy, the development and utilization of sustainable energy resources have emerged as a critical global issue [1,2,3]. Geothermal energy, recognized as a clean, efficient, and renewable resource, offers considerable potential for development [4,5,6]. In particular, Hot Dry Rock (HDR) geothermal systems, known for their high temperatures and substantial energy storage deep within Earth’s crust, represent a promising geothermal resource [7,8]. It is estimated that the geothermal energy content of HDR is 800 times that of hydrothermal resources and 300 times that of the total energy from fossil fuels [9]. Effective exploitation of HDR resources can not only help alleviate the energy crisis but also reduce environmental pollution and greenhouse gas emissions, offering significant environmental and social benefits. The creation of sufficiently interconnected channels within the reservoir is crucial for commercial-scale heat extraction from HDR geothermal systems [10]. During the geothermal extraction process, the introduction of low-temperature fluids, such as water or liquid nitrogen, into the rock strata cools the high-temperature HDR reservoirs and generates thermal stress, inevitably damaging the reservoirs. This reduces the fracturing stress during hydraulic fracturing and enhances reservoir permeability [11], which significantly impacts the extraction efficiency of HDR [12]. However, different cooling rates have varying impacts on the physical and mechanical properties of high-temperature rocks, thereby affecting the extent of damage. Therefore, a thorough understanding of the effects of cooling rates on the physical and mechanical properties of rocks is particularly crucial for the production efficiency of geothermal engineering.

HDR reservoirs are characterized by high temperatures, which significantly alter the properties of rocks [13,14,15,16]. The effects of heating on rock properties have been well-documented, with changes in physical and mechanical properties primarily due to the evaporation of pore water [17,18,19], decomposition of mineral components [20,21,22,23], and transformations in the crystal structures within mineral grains [24,25,26]. The contact between the high-temperature rocks of HDR reservoirs and external cold media during extraction induces thermal stress due to cooling, thus affecting the rock properties. Water cooling, a widely used method, has been extensively studied in recent years. Wu et al. [27] found that heated granite subjected to water cooling during Brazilian split tests exhibited severe thermal damage, resulting in lower tensile strength (*σ*_t_) compared to samples that were naturally cooling. Xue [28] explored the impact of water cooling on rock crack formation and morphology through the thermal/hydro/mechanical coupling model and hydraulic fracturing experiments on heated granite. The results indicated that water cooling significantly reduces *σ*_t_ and elastic modulus (*E*) of rocks, especially under high temperatures, promoting crack formation and expansion, thus leading to a more complex fracture network. Zhu et al. [29] observed that the density and mechanical strength of granite decreased with increasing cycles of cyclic water cooling, with notable reductions in uniaxial compressive strength (UCS) and *E* after 30 cycles. Scanning electron microscopy (SEM) results suggested that the significant increase in microcracks was the primary cause of the deterioration in the physical and mechanical properties of granite. Further studies by Cui et al. [30] showed that increasing the number of heating/cooling cycles gradually decreased the *P*-wave velocity (*V*_p_) of granite, with a reduction in mass and an increase in volume, attributed to structural changes caused by the thermal expansion and contraction of mineral particles due to moisture evaporation under high temperatures. Moreover, Wang et al. [31] investigated the effects of different water cooling durations under varying temperatures on the fracture performance of granite. The results indicated that the influence of water cooling on fracture performance was slightly less than that of temperature. At the same temperature, the fracture toughness first decreased, then increased, and finally, decreased again as the water cooling duration extended. Fu et al. [32] analyzed the impact of water cooling treatment on the mechanical properties and damage behavior of clayey sandstone, indicating that *E*, cohesion, and internal friction angle of clayey sandstone significantly decreased, with the extent of damage closely related to the temperature and number of water cooling cycles. These findings collectively highlight the profound impact of water cooling on the structural integrity and mechanical properties of rocks, providing robust support for the extraction of HDR.

When the environmental temperature of reservoir rocks changes abruptly, thermal stress is generated within the rocks, determined by the temperature difference between the rock and the cooling medium. Therefore, some pioneering studies have attempted to use cooling media at lower temperatures to enhance the extraction efficiency of HDR [33]. Liquid nitrogen (LN_2_), with a temperature as low as −196 °C under normal atmospheric conditions, creates a substantial temperature difference when introduced into HDR reservoirs, thereby altering the physical and mechanical properties and pore structures of rocks [34]. Su et al. [35] used LN_2_ to cool heated marble, observing a significant increase in porosity, while l *V*_p_, UCS, and *E* showed a decreasing trend, primarily due to thermal damage incurred during rapid cooling. Similarly, Ding et al. [36] found that liquid nitrogen cooling significantly increased the total porosity of heated limestone, especially after high-temperature treatment. The increase in internal porosity of the rocks reduces their mechanical properties. Additionally, Zhou et al. [37] discovered that LN_2_ cooling significantly reduces the *σ*_t_ of rocks, with the degree of reduction varying at different temperatures. Before reaching a temperature threshold, the effect of liquid nitrogen cooling was found to be superior to that of water cooling; beyond this threshold, its effectiveness was less than that of water cooling. However, when temperatures exceeded 200 °C, liquid nitrogen cooling resulted in granite exhibiting more shear or mixed-mode fractures during failure, increasing the roughness of the fracture surface. Xi et al. [38] conducted Hopkinson bar tests on rocks after LN_2_ cooling and found that the fragments had a higher fractal dimension, indicating that the rapid cooling by LN_2_ caused a swift release of internal stresses, resulting in numerous small and complex fragments and varying degrees of damage within the rocks. Since *V*_p_ can effectively reflect the damage in rocks, Sun et al. [39] observed attenuation in *V*_p_, amplitude, and main frequency with each LN_2_ cooling cycle, where wave velocity attenuation could reach up to 85.01%. These studies clearly demonstrated that LN_2_ cooling treatment has profound effects on the physical and mechanical behavior of various rocks. It is evident that not only the choice of cooling medium but also the rate at which cooling is applied plays a pivotal role in determining the extent and nature of rock damage in HDR systems. This understanding is essential for optimizing extraction processes and improving the overall efficiency of geothermal energy systems.

Existing research has extensively investigated the impacts of heating/cooling cycles on the physical and mechanical properties of rocks; some related research is summarized in Table 1. Furthermore, some researchers have observed significant differences in the dynamic mechanical properties of sandstone after heating under various cooling treatments (natural cooling, water cooling, and LN_2_ cooling) [40]. This indicates that the rate of cooling can significantly affect the physical and mechanical properties of rocks. It can be inferred that due to the cooling shock from cooling media during the extraction process of Hot Dry Rock (HDR) reservoirs, different cooling rates can also significantly alter the physical and mechanical properties of the reservoir rocks. However, current research on the effects of different cooling rates on heated granite, a common matrix rock in HDR geothermal reservoirs, remains relatively scarce. Although some studies have explored the impacts of natural cooling, water cooling, and liquid nitrogen cooling on the physical and mechanical properties of heated rock, systematic studies on cyclic liquid nitrogen cooling are still limited. In particular, research comparing the effects of cyclic liquid nitrogen cooling with other cooling methods, such as natural cooling, water cooling, and single liquid nitrogen cooling, on the physical and mechanical properties of heated granite is still not comprehensive. Therefore, this study aims to investigate the deteriorative effects of different cooling rates (cooling in air, cooling in water, cooling in LN_2_, and cyclic cooling in LN_2_) on the physical and mechanical properties of heated granite. We designed a series of physical property tests and mechanical experiments on granite heated to different temperatures and subsequently subjected to various cooling treatments. The study first analyzed changes in granite density, *V*_p_, UCS, *σ*_t_, and *E* after different treatments. Then, the microstructural features of the samples were examined using NMR non-destructive testing and SEM. Finally, the damage mechanisms of heated granite treated with different cooling methods were analyzed. This research can provide theoretical insights for the extraction of geothermal resources.

## 2. Materials and Methods

**Figure 1:**
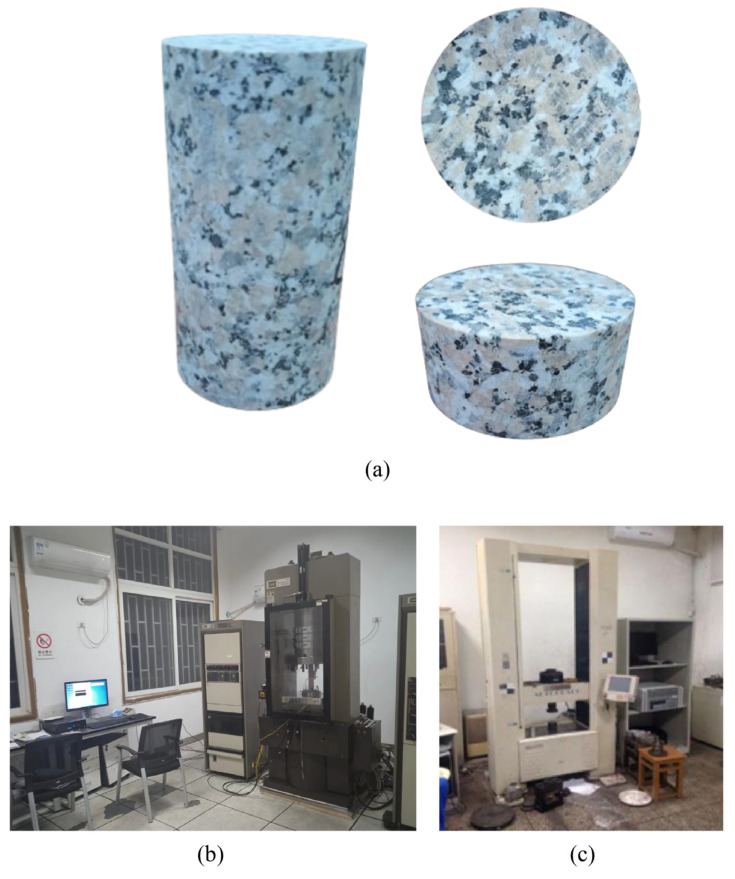
Samples and equipment. (**a**) Specimens for uniaxial compression strength experiment and Brazilian split tests. (**b**) MTS815 experimental apparatus. (**c**) AG-250kN IS Electronic Precision Material Testing Machine.

Granite, as a typical rock type in Hot Dry Rock, was selected as the experimental material in our study. This study utilized granite samples sourced from Rizhao, Shandong Province, China, for subsequent experiments. Samples were selected from large rock masses that exhibited no visible cracks or signs of weathering. In accordance with the International Society for Rock Mechanics (ISRM), a rock-cutting machine was used to cut the rocks into standard specimens of 50 mm diameter × 100 mm height and disc specimens of 50 mm diameter × 25 mm height. All specimens were polished on both ends to ensure end flatness of less than 0.02 mm, as shown in Figure 1a. The 50 × 100 mm^2^ specimens were used for wave velocity tests and uniaxial compressive strength tests, while the 50 × 25 mm^2^ specimens were used for Brazilian split tests to determine tensile strength. MTS 815 Flex Test GT rock mechanics system produced in the United States and AG-250kN IS Electronic Precision Material Testing Machines produced in Japan were employed for the uniaxial compressive strength experiment and Brazilian split tests, respectively, as illustrated in Figure 1b,c. For MTS 815, the maximum axial load reaches 2800 kN, the maximum peripheral pressure is 80 MPa, the maximum pore water pressure is 80 MPa, and the temperature is up to 200 °C. The maximum axial load of the AG-250kN IS is 250 kN, the tester stiffness is 15 GN/m, and the range of the loading rate is 0.0005–1000 mm/s [41,42].

After processing, based on existing research, a heating rate of 2 °C/min was selected to heat the temperature to the target value to avoid thermal shock [5]. The target values in this study are 200 °C and 300 °C. Once the target temperature was reached, the temperature was maintained for 10 h to ensure uniform internal heating of the samples. After heating, the samples were subjected to four types of cooling treatments: cooling in air (CA), cooling in water (CW), cooling in liquid nitrogen (CL), and c cycle cooling in liquid nitrogen (CCL) for 5 cycles, as detailed in Table 2. After the cooling treatment, the granite specimens were dried again in an air-drying oven, and then physical and mechanical tests were performed. We first tested the density and wave speed of the granite standard pieces before and after the heating/cooling treatment. Before conducting mechanical experiments, we tested the pore structure of the heated granite samples after cooling treatments. Then, uniaxial compressive strength tests and tensile strength tests were conducted after the heating/cooling treatments. After the mechanical experiments, samples were sliced from the fracture surfaces for SEM testing to obtain the microstructural damage characteristics under different cooling methods.

## 3. Results

### 3.1. Physical Properties

#### 3.1.1. Density

Changes in the mass and volume of granite can reflect alterations in its internal structure to some extent. High temperatures and rapid cooling can cause changes in the internal pore structure of granite, such as the merging of pores or the formation of new pores, which also affect the rock’s density. During the heating and rapid cooling process, the detachment of boundary particles in granite may occur, reducing the mass of the granite and thereby decreasing its density. Therefore, this paper first calculates the mass loss rate, volumetric expansion rate, and density of granite after treatment with different cooling methods. The formulas used for these calculations are as follows:

(1) $$\Delta m=\frac{m_{1}-m_{0}}{m_{0}}\times 100\%$$

(2) $$\Delta V=\frac{V_{1}-V_{0}}{V_{0}}\times 100\%$$

(3) $$\rho =\frac{m_{1}}{V_{1}}$$

where *m*_0_ and *m*_1_ represent the mass of the granite sample before and after the heating/cooling treatment, respectively, g; *V*_0_ and *V*_1_ represent the volume of the granite sample before and after the heating/cooling treatment, respectively, cm^3^.

**Figure 2:**
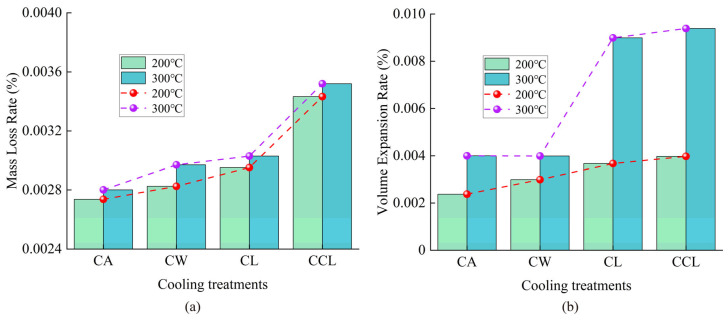
Changes in mass loss rate and volume expansion rate of granite under different heating temperatures and cooling treatments. (**a**) Results of the temperature at 200 °C. (**b**) Results of the temperature at 300 °C.

At temperatures of 200 °C and 300 °C, both the mass loss rate and the volumetric expansion rate of granite show significant changes with varying cooling methods, as illustrated in Figure 2. The CCL treatment results in higher mass loss rates and volumetric expansions compared to the other three methods. The reasons for these trends can be attributed to two main factors. On the one hand, moisture evaporation plays a role, as granite contains certain amounts of bound and free water. During the heating process, a considerable amount of free water and some bound water evaporate in gaseous form, which is a key factor in the mass loss of granite. On the other hand, the detachment of surface particles is due to differences in the coefficients of thermal expansion among the various mineral components of granite. Heating causes uneven expansion within the rock’s internal minerals, followed by uneven contraction during subsequent cooling. This uneven thermal expansion and contraction weaken the binding ability between rock particles, potentially leading to the detachment of surface particles. With increased cooling rates, granite undergoes more intense uneven contraction, potentially leading to greater surface particle detachment and, thus, larger mass loss.

**Figure 3:**
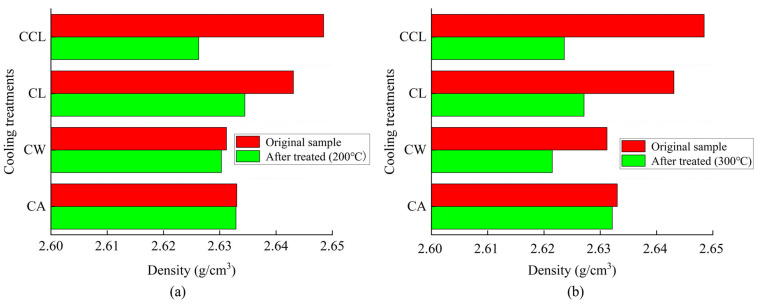
Density changes of granite after different cooling methods. (**a**) Results of the temperature at 200 °C. (**b**) Results of the temperature at 300 °C.

Changes in the density of granite following different cooling treatments are shown in Figure 3. At 200 °C, the density of granite remains virtually unchanged after CA and CW treatments, whereas it changes significantly after CL and CCL treatments. At 300 °C, the density of granite changes after CW, CL, and CCL treatments, with no significant change following CA treatments. This indicates that the greater the temperature difference during cooling treatment, the more likely it is that the density of granite will significantly decrease after treatment. The mass loss rates, volumetric expansion rates, and density of high-temperature granite all change after undergoing different cooling treatments, suggesting that the internal structure of the rock may be affected, which could further influence its wave speed characteristics. Therefore, we further analyze the changes in the *V*_p_ of granite before and after cooling treatment.

#### 3.1.2. Effect of Different Cooling Treatments on *V*_p_

**Figure 4:**
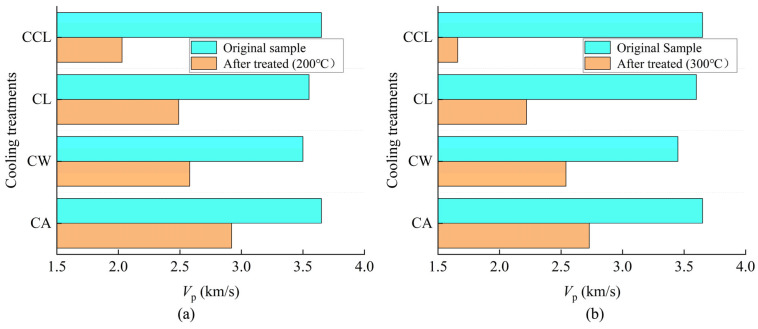
Changes of *V*_p_ of granite treated with different cooling methods. (**a**) Results of the temperature at 200 °C. (**b**) Results of the temperature at 300 °C.

As a non-destructive testing method, measuring *V*_p_ is highly sensitive to internal rock features such as cracks and porosity, thus effectively reflecting the internal damage state of the rock and indirectly estimating its mechanical strength. As shown in Figure 4, under conditions of 200 °C and 300 °C, *V*_p_ after all four cooling treatments exhibited significant changes. At 200 °C, compared to pre-treatment speeds, the *V*_p_ of samples after CA, CW, CL, and CCL cooling treatments decreased by 20%, 26.3%, 29.9%, and 44.4%, respectively. Similarly, at 300 °C, *V*_p_ samples after CA, CW, CL, and CCL cooling treatments decreased by 25.2%, 26.4%, 38.3%, and 54.5%, respectively, compared to before treatment. This is because as the cooling rate increases, the uneven thermal stress within the rock intensifies, leading to the formation and expansion of microcracks, which, in turn, affects the rock’s density and internal structure, resulting in a reduction in wave speed, as described in Section 3.1.1. The density of granite is also significantly affected by different cooling treatments.

### 3.2. Mechanical Properties

#### 3.2.1. Effect of Different Cooling Treatments on UCS

**Figure 5:**
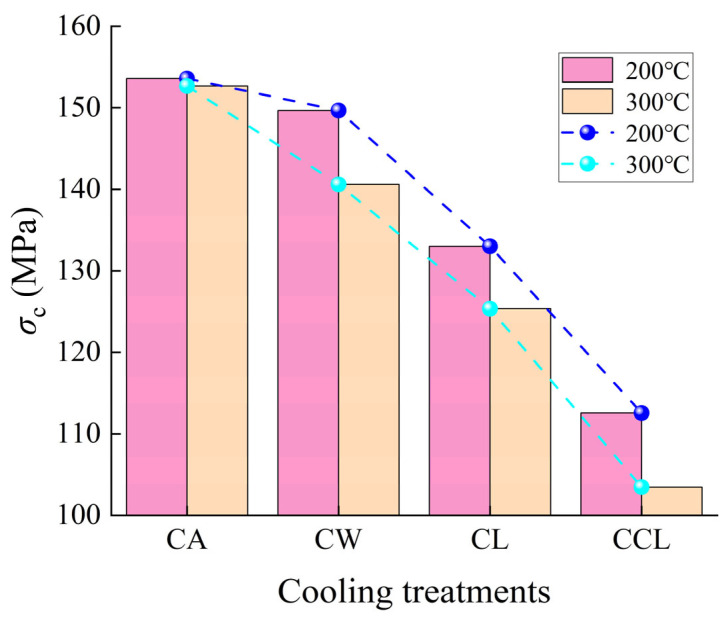
Uniaxial compressive strength of granite after different cooling treatments.

As shown in Figure 5, the UCS (uniaxial compressive strength) of granite generally decreases with increasing cooling rates. At 200 °C, compared to the UCS of samples after CA (cooling in air) treatment, the UCS of samples after CW (cooling in water), CL (cooling in liquid nitrogen), and CCL (cycle cooling in liquid nitrogen) treatments decreased by 2.6%, 13.4%, and 26.7%, respectively. Similarly, at 300 °C, compared to the UCS of samples after CA treatment, the UCS of samples after CW, CL, and CCL treatments decreased by 7.9%, 17.9%, and 32.2%, respectively. It is evident that different heating temperatures and cooling rates significantly affect the UCS of granite, particularly after CCL treatment, where the reduction in UCS is especially pronounced. This is due to the differences in the coefficients of thermal expansion of the various mineral particles composing the granite. During the cooling process, an increase in the cooling rate exacerbates the mismatched deformation between different mineral particles and also increases the temperature gradient between the inner and outer layers of the granite, thus generating higher thermal stresses, which further intensify the internal damage to the granite, reducing its mechanical strength.

#### 3.2.2. Effect of Different Cooling Treatments on *σ*_t_

**Figure 6:**
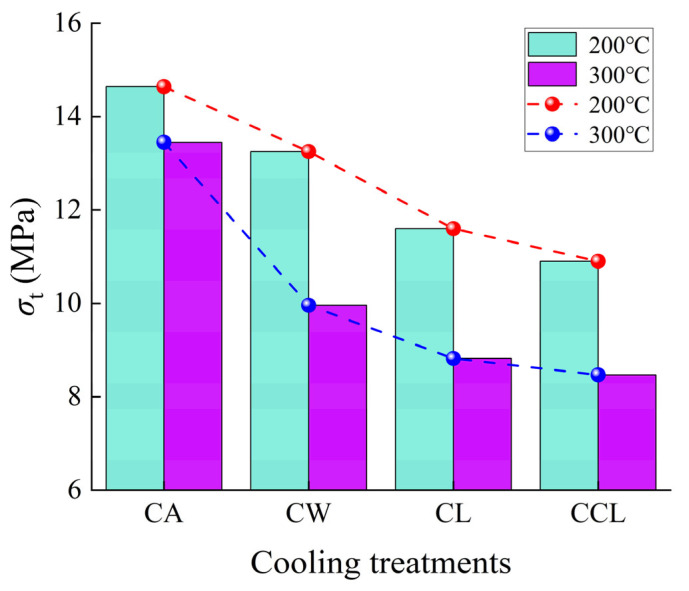
Changes in the tensile strength of granite under different cooling methods.

As shown in Figure 6, the *σ*_t_ (tensile strength) of granite samples tends to decrease with increasing cooling rates. Particularly at 300 °C, the *σ*_t_ of granite samples subjected to rapid cooling significantly decreases. Specifically, at 200 °C, compared to the *σ*_t_ of samples after CA, the *σ*_t_ of samples after CW, CL, and CCL treatments decreased by 9.5%, 20.8%, and 25.5%, respectively. Similarly, at 300 °C, compared to the *σ*_t_ of samples after CA, the *σ*_t_ of samples after CW, CL, and CCL treatments decreased by 25.9%, 34.4%, and 37%, respectively. The reduction in *σ*_t_ is mainly due to various degrees of internal damage caused by temperature differences. During rapid cooling, the sharp decrease in temperature causes rapid contraction of the mineral grains within the rock. Due to the heterogeneity of mineral composition, differences in the coefficients of thermal expansion among different minerals lead to uneven distribution of internal stresses, thereby causing the formation and expansion of microcracks, which, in turn, reduces the *σ*_t_ of granite.

#### 3.2.3. Effect of Different Cooling Treatments on *E*

**Figure 7:**
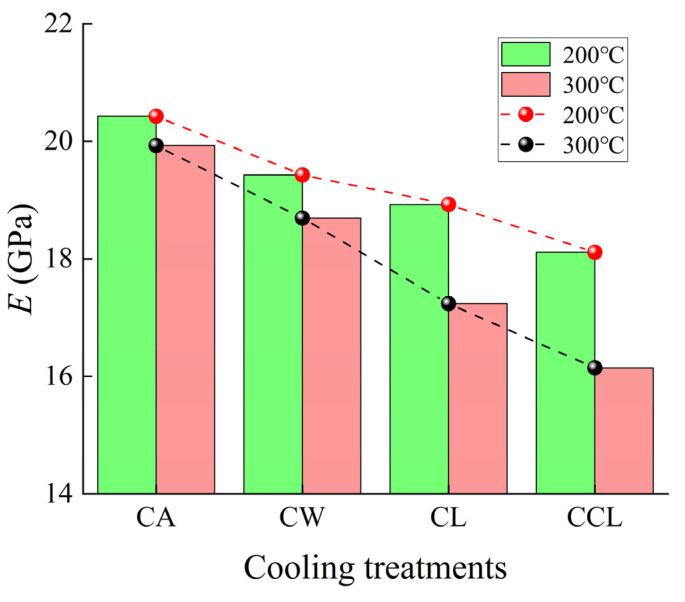
Changes of elastic modulus of granite under different cooling methods.

*E* represents the rock’s ability to resist deformation. As shown in Figure 7, except for natural cooling, the *E* of granite at 200 °C is higher than at 300 °C. With increasing cooling rates, the *E* of granite shows a decreasing trend. At 200 °C, compared to the *E* of samples after CA, the *E* of samples after CW, CL, and CCL treatments decreased by 4.9%, 7.3%, and 11.3%, respectively. At 300 °C, compared to the *E* of samples after CA, the *E* of samples after CW, CL, and CCL treatments decreased by 6.2%, 13.5%, and 19%, respectively. At lower cooling rates, the internal temperature distribution of granite is relatively uniform, producing relatively minor thermal stresses; therefore, the development of microcracks inside the rock is slow, and changes in the granite’s *E* are minimal. However, rapid cooling can cause a significant temperature difference between the surface and the interior of the granite, leading to substantial thermal stress, which in turn rapidly generates and expands internal microcracks, thereby reducing the rock’s *E*. Since *E* is closely related to the integrity of its internal structure, an increase in microcracks and porosity means the internal structure of the rock becomes more loose and fragile. Therefore, we further analyzed the changes in the micro-porous structure of granite before and after different cooling treatments.

### 3.3. Microstructural Features

#### 3.3.1. Effect of Different Cooling Treatments on Heated Granite Pore Size

**Figure 8:**
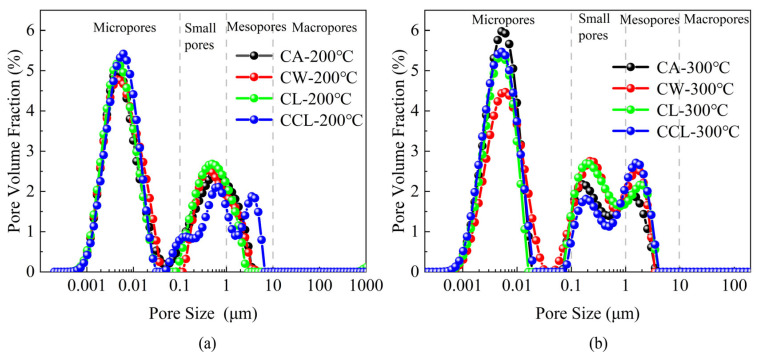
Pore size distribution of heated granite samples under different cooling methods. (**a**) Results of the temperature at 200 °C. (**b**) Results of the temperature at 300 °C.

We used nuclear magnetic resonance (MacroMR12-150H-I) to test the porosity of granite. Drawing on current research on micro-porous structures, we have categorized the porosity of granite into large pores (>10 μm), mesopores (1–10 μm), small pores (0.1–1.0 μm), and micropores (<0.1 μm), as shown in Figure 8. The porosity of granite is primarily composed of micropores, small pores, and mesopores. With increasing cooling rates, the proportion of micropores and small pores in granite gradually increases, and there is a clear trend of increasing pore size. For instance, at 200 °C, the proportion of small pores in granite subjected to cyclic liquid nitrogen treatment is slightly lower than that observed under the other three treatments, but the volume proportion of these pores is significantly larger, notably higher than the mesopore proportion under other treatments. Similarly, at 300 °C, the mesopore volume proportion in granite samples treated with cyclic liquid nitrogen is the largest. Specifically, at 200 °C, the proportions of micropores in samples after CA, CW, CL, and CCL treatments are 3.95%, 3.24%, and 3.62%, respectively; small pores are 71.11%, 73.39%, and 73.37%, respectively; and mesopores are 24.94%, 23.37%, and 53.42%, respectively. At 300 °C, the proportions of micropores in samples after CA, CW, CL, and CCL treatments are 6.14%, 5.23%, and 4.97%, respectively; small pores are 66.20%, 64.39%, 45.96%; and mesopores are 27.66%, 30.38%, 49.08%. This indicates that an increase in cooling rate facilitates the formation of small pores and promotes the transformation of small pores into mesopores in heated granite, thereby altering the physical and mechanical properties of the rock and causing varying degrees of reduction.

#### 3.3.2. Effect of Different Cooling Treatments on Porosity

**Figure 9:**
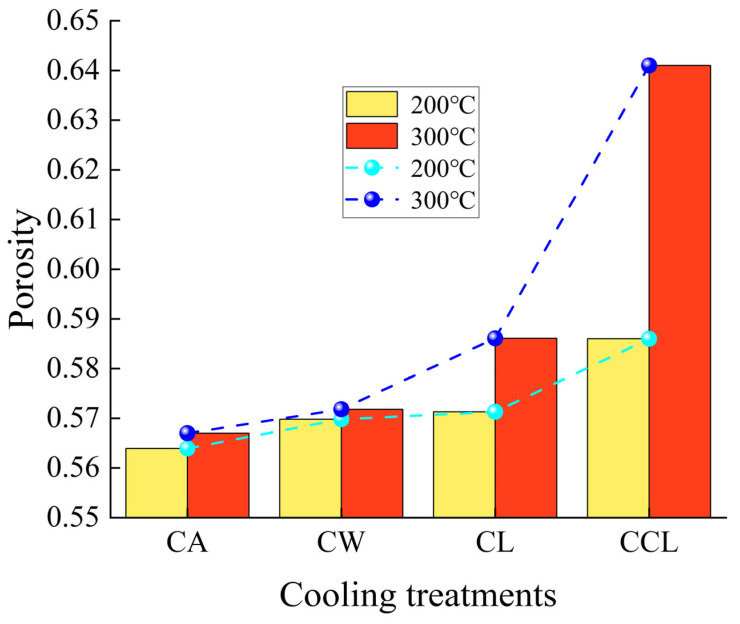
Changes in granite porosity under different cooling methods.

Porosity is an important parameter reflecting the pore structure characteristics of rocks, significantly influencing their physical properties, mechanical strength, and permeability. Porosity can be defined as the ratio of pore volume to the total volume, known as the porosity rate, as shown in Figure 9. With increasing cooling rates, the porosity of granite shows an upward trend, with more significant changes in porosity rate observed at 300 °C. This is because rapid cooling can be considered a form of cold shock to the rock. Since there is a greater temperature difference between the rock heated to 300 °C and LN_2_, undergoing LN_2_ treatment can generate a greater cold shock. This shock can lead to rapid development and interconnection of microcracks within the rock, forming larger pores. Additionally, the liquid-to-gas phase transition of LN_2_ may also generate pressure within microcracks and pores of rock, promoting the connectivity of the pores.

#### 3.3.3. Effect of Different Cooling Treatments on the Micromorphology of Heated Granite

**Figure 10:**
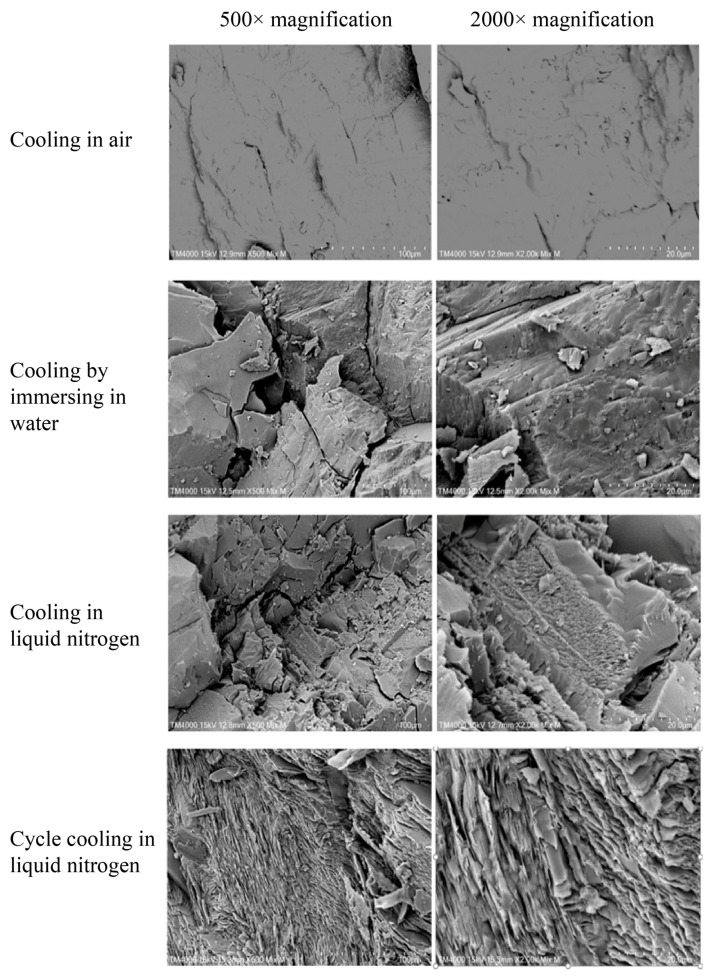
Morphology of fracture surfaces of heated granite under different cooling treatments at 200 °C.

**Figure 11:**
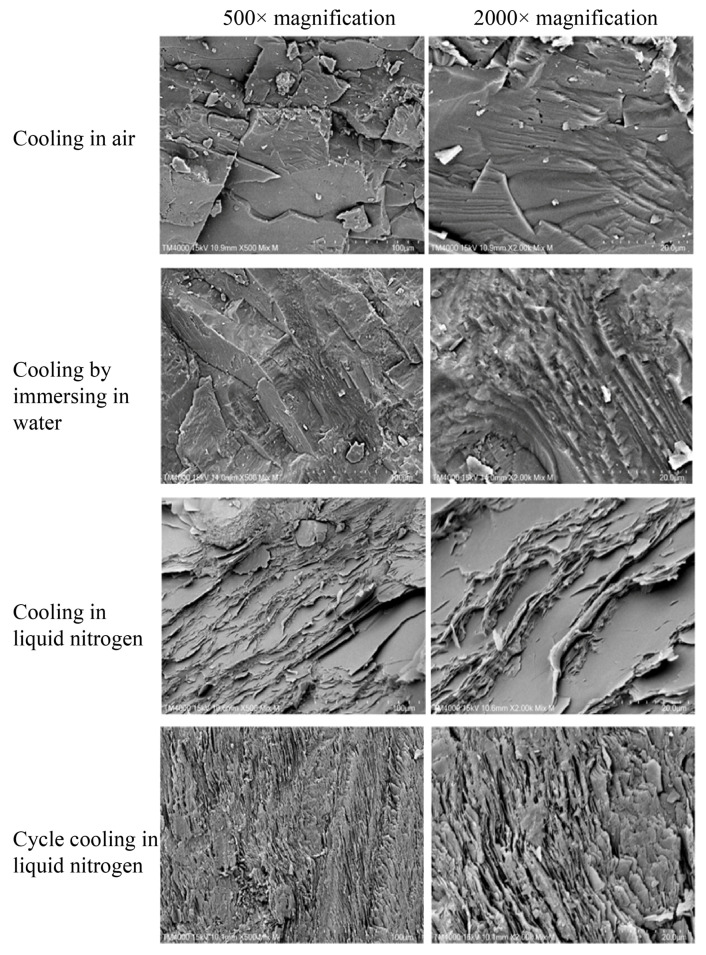
Morphology of fracture surfaces of heated granite under different cooling treatments at 300 °C.

From the results shown in Figure 10 and Figure 11, it can be observed that with increasing cooling rates, the microstructure of granite gradually transitions from dense to loose, and the characteristics of the fracture surfaces reflect a transition from brittle to ductile failure. After CA treatment, the fracture surfaces of granite samples are relatively smooth, with large blocky structures at 500× magnification and a relatively sparse distribution of crack networks. When granite samples undergo CW treatment, the blocky structures are reduced in size at 500× magnification, and the crack network becomes denser. After CL treatment, the granite samples display visible cracks at 500× magnification, and multiple pores and fragments appear at 2000× magnification. Following CCL treatment, distinct through-going cracks can be observed at 500× magnification, with significantly widened crack openings. At 2000× magnification, the fracture surface shows a net-like distribution of cracks after CCL treatment, with microcracks significantly denser than those seen with the other three cooling treatments. These results of SEM provide a visual reference for understanding the impact of different cooling methods on the microstructure of granite. It can be seen that cooling treatments can alter the microstructure of heated granite, especially CCL treatment, which significantly increases the roughness of the fracture surfaces, effectively deteriorating the fine microstructure of the granite.

## 4. Discussion

### 4.1. Damage Factor

It has been demonstrated that the *V*_p_ of rocks can provide a significant indication of their damage situation [43]. As suggested by some researchers, a damage factor is defined in this research, which is related to the *V*_p_, shown as:

(4) $$D=1-{\left(\frac{V_{post}}{V_{before}}\right)}^{2}$$

where *D* is the damage factor, and *V*_post_ and *V*_before_ are the *P*-wave velocities of the heat/cooling-treated specimens and untreated specimens, respectively.

**Figure 12:**
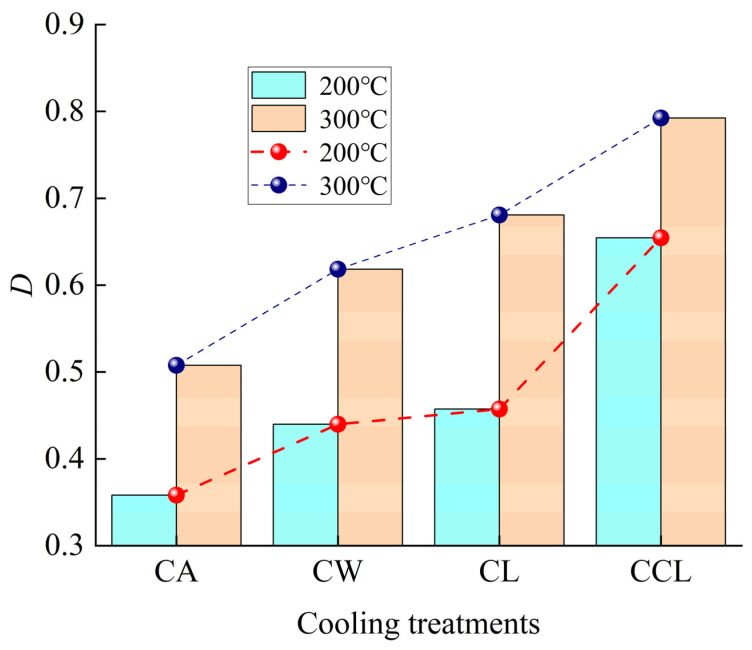
Changes in *D* of heated granite under different cooling methods.

Figure 12 shows the trend of *D* in granite following different cooling treatments. According to the results shown in the figure, different cooling methods significantly affect the extent of damage in granite. As the cooling rate increases, *D* at different temperatures shows an upward trend. At 200 °C, the *D* of granite after CA, CW, CL, and CCL treatments are, respectively, 0.3582, 0.4397, 0.4274, and 0.6544. At 300 °C, the *D* of granite after CA, CW, CL, and CCL treatments are, respectively, 0.5077, 0.6184, 0.6809, and 0.7924. Comparisons reveal that at heating temperatures of 200 °C and 300 °C, the increase in the damage variable after CCL treatment is significantly greater, being 45.3% and 35.9% higher than after CA treatment, respectively. This may be due to the rapid contraction of any existing microcracks or pores within the granite under the extremely low temperatures of CCL treatment, causing stress concentration, which can rapidly lead to material damage in localized areas. Generally, *D* can reflect the internal damage of rock specimens with temperature and different cooling treatments, consistent with the experimental results. As described in Section 3.2.1, Section 3.2.2 and Section 3.2.3, the mechanical properties of granite have been significantly weakened.

### 4.2. Damage Mechanism of Heating and Different Cooling

In this section, we aim to reveal the mechanisms of thermal cracking from both perspectives of heating and different cooling treatments. The damage to granite in this study is primarily caused by two factors: high temperatures during the heating process, causing rock damage, and thermal stress from cooling treatments, causing additional rock damage. Granite is composed of various mineral grains, such as quartz, feldspar, and mica, arranged in a disordered manner within the rock. After the samples are heated, incompatible deformations between these minerals, along with thermal stress caused by the evaporation of free and bound water, lead to the formation and expansion of microcracks. Increasing the temperature gradually increases the thermal stress within the rock, providing energy for the expansion of these microcracks. Therefore, in the temperature changes set in this study, granite samples heated to 300 °C exhibited a more pronounced decrease in wave speed and an increase in porosity. This further leads to a larger damage factor in granite treated at 300 °C.

Moreover, when high-temperature granite undergoes rapid cooling, the intense cold shock generates a considerable temperature gradient, further increasing thermal stress. According to research by Kim et al. [44], when heated rocks come into contact with a coolant, the temperature gradient near the surface of the sample is much higher than near the center of the sample. This creates tensile stress in the external regions of the sample and compressive stress internally. Granite, being a typical brittle material, is more susceptible to damage under tensile stress than under compressive stress. Thus, the tensile stress generated in the external areas of the sample is the primary cause of microcrack expansion and the subsequent deterioration of the rock’s properties.

To quantitatively describe the impact of thermal shock on high-temperature granite, Collin et al. proposed a formula for calculating the tensile stress generated in brittle materials due to thermal shock:

(5) $$\sigma_{t}^{T}=\frac{E\alpha \Delta T}{1-\mu }f_{h}$$

(6) $$f_{h}=\frac{1}{\frac{ak}{hr}+b-ce^{{-}_{hr}^{dk}}}$$

where *k*, *E*, *μ*, *α*, and *r* represent the thermal conductivity, elastic modulus, Poisson ratio, thermal expansion coefficient, and radius of the sample, respectively; Δ*T* and *h* represent the temperature difference and the heat transfer coefficient between the sample and the coolant, respectively; the coefficient *a*, *b*, *c,* and *d* for the brittle material can be obtained from Collin and Rowcliffe.

For granite heated to a specific temperature, the constants *k*, *E*, *μ*, *α*, and *r* remain unchanged. Therefore, according to Equation (5), the tensile stress is determined by Δ*T* and *h.* The heat transfer coefficients, *h*, for water and liquid nitrogen exhibit significant differences. For cylindrical granite, this coefficient ranges from 200 to 1400 W/m^2^ K during water cooling and escalates to between 3000 and 39,000 W/m^2^ K during liquid nitrogen cooling [45]. Collin and Rowcliffe point out that *f*_h_ increases monotonically with the heat transfer coefficient h [45]. Therefore, this results in higher values of *f*_h_ for the LN_2_ cooling condition compared to the CW treatment condition. Moreover, due to the markedly low temperature of LN_2_ (−196 °C), the temperature difference Δ*T* between the heated granite and the LN_2_ far exceeds that between the heated granite and water. Therefore, compared to CW treatment, the tensile stress generated internally by CL treatment is higher, resulting in more pronounced damage to the samples treated with LN_2_. This also explains the reason that heated granite shows more significant deterioration in density, *V*_p_, UCS, *E*, and *σ*_t_, as well as rougher fracture surfaces after CL and CCL treatments compared to samples treated with CA and CW treatments. These results indicate a substantial decline in the physical and mechanical properties of granite treated with CL treatment, particularly after CCL treatments. Therefore, in future HDR extraction processes, LN_2_ fracturing or cyclic LN_2_ fracturing holds significant potential, likely enhancing the production efficiency of HDR.

## 5. Conclusions

Investigating the physical and mechanical properties of heated rock under different cooling methods has significant implications for the exploitation of HDR. Ultrasonic testing, uniaxial strength compression experiments, Brazilian splitting tests, nuclear magnetic resonance (NMR), and SEM were conducted on heated granite after different cooling methods, including cooling in air, cooling in water, cooling in liquid nitrogen, and cycle cooling in liquid nitrogen in this study. The special conclusions are as follows:

(1) As the cooling rate increases, the mass loss rate and volumetric expansion rate of granite gradually increase, while the density and *V*_p_ of granite tend to decrease.
(2) With an increasing cooling rate, the UCS, *σ*_t_, and *E* of heated granite show an overall decreasing trend. Samples subjected to CCL treatment not only exhibit a more pronounced decline in mechanical properties but also a higher degree of damage. These results can provide a theoretical reference for the liquid nitrogen cycle fracturing process in geothermal production, thereby optimizing fracturing parameters and improving geothermal production efficiency.
(3) Both the pore size and porosity of granite tend to increase with an increasing cooling rate, and the morphology of the fracture surfaces transitions from smooth to rough, forming more complex surface cracks.

## Figures and Tables

**Table 1:** Related indicators in the published literature.

	Granite	Preparation Method	Physical and Mechanical Characteristics
Ref. [27]	Changsha gray granite	Slow heating followed by water or air cooling	Tensile strength
Ref. [29]	Gray and fine-grained granite	Cycle heating and water-cooling	Volume, mass, density, UCS, and E
Ref. [31]	Granite from Hubei province, China	Water cooling after heated	Fracture toughness
Ref. [37]	Granite from Rizhao, China	Liquid nitrogen cooling and water cooling	Tensile strength

**Table 2:** Summary of different heated temperature and cooling methods of granite.

Number	Heated Temperature	Cooling Methods
CA-200	200	CA
CA-300	300	CA
CW-200	200	CW
CW-300	300	CW
CL-200	200	CL
CL-300	300	CL
CCL-200	200	CCL
CCL-300	300	CCL

## Data Availability

The original contributions presented in the study are included in the article; further inquiries can be directed to the corresponding author.
